# Supplementary material for: Persistent expression of activation markers on Mycobacterium tuberculosis-specific CD4 T cells in smear negative TB patients
Source: PLoS One. 2022 Aug 30;17(8):e0271234. doi: 10.1371/journal.pone.0271234 (PMC9426896; doi:10.1371/journal.pone.0271234)
Supplement: S1 File — (DOCX) [file pone.0271234.s002.docx]

**Response to editors requirements**

**Title: Persistent expression of activation markers on Mycobacterium tuberculosis-specific CD4 T cells in smear negative TB patients**

PONE-D-22-05355

Ahmed Esmael^,^, Adane Mihret, Tamrat Abebe, Daniel Mussa , Sebsibe Neway, Joel Ernst, Jyothi Rengarajan, Liya Wassie, Rawleigh Howe

We would like to express our gratitude to editors for providing us with helpful feedbacks that helped us to enhance our paper so that it could be published in reputable journal. Based on the reviewers and journal requirements we modify our manuscript and cover page section. We   responded to comments as follows:

1. **Editors Requirements**

**1. *Data Availability statement****:*  The manuscript and its supporting information files contain all necessary data. Our data was uploaded to a public repository and may be found at https://figshare.com/account/home, doi 10.6084/m9.figshare.19753798. Additionally, the data is uploaded as supporting files.

**With regards**

**Ahmed Esmael**
