# Supplementary material for: Persistent expression of activation markers on Mycobacterium tuberculosis-specific CD4 T cells in smear negative TB patients
Source: PLoS One. 2022 Aug 30;17(8):e0271234. doi: 10.1371/journal.pone.0271234 (PMC9426896; doi:10.1371/journal.pone.0271234)
Supplement: Attachment — Submitted filename: Ahmed response for editor final.docx [file pone.0271234.s004.docx]

**Response to editors requirements**

**Title: Persistent expression of activation markers on Mycobacterium tuberculosis-specific CD4 T cells in smear negative TB patients**

PONE-D-22-05355

Ahmed Esmael^,^, Adane Mihret, Tamrat Abebe, Daniel Mussa , Sebsibe Neway, Joel Ernst, Jyothi Rengarajan, Liya Wassie, Rawleigh Howe

We would like to express our gratitude to editors for providing us with helpful feedbacks that helped us to enhance our paper so that it could be published in reputable journal. Based on the reviewers and journal requirements we modify our manuscript and cover page section. We   responded to comments as follows:

A. **Journal Requirements**
1. Please review your reference list to ensure that it is complete and correct.

**Response**: Thank you for your comments. Deeply apology for the citation problems in the discussion part. I do not know why such issues as far as I used endnote reference citation tool. I made revision for some references which needs some correction, especially related with formatting, completeness and citation issues in the discussion part.

B. **Response for Reviewer #1**
1. They may need to include more references (regarding immune responses CD4+or CD8+ T cells before and after anti-TB therapeutics in TB patients) from other labs in the similar field. I have no more other comments.

**Response**: We incorporated and acknowledged similar works in the introductory part.

C. **Response for Reviewer #2**
1. The arrangement of graphs is not very orderly, and the font size is inconsistent. Please readjust them.

**Respons**e: We made modification for font size and arrangement for all graphs in the manuscript and tried to re-organize the order in the result section.

2. Please simplify the discussion section according to the results and the main idea of the article.

**Response**: We made revision in the discussion part without affecting the main finding.

**With regards**

**Ahmed Esmael**
